# Assessing disaster resilience in mountain villages using an improved DPSIR-A framework and multi-model machine learning

**DOI:** 10.1038/s41598-026-57866-8

**Published:** 2026-06-13

**Authors:** Liuqin Yan, Li Zhang, Yaofan Ye, Baoli Han

**Affiliations:** 1 School of Architecture, Southwest Minzu University, Chengdu, 610041 Sichuan China; 2 School of Architecture and Urban Planning, Hunan University, Changsha, 410006 China

**Keywords:** Environmental sciences, Environmental social sciences, Natural hazards

## Abstract

Amid the dual challenges of global climate change and frequent geological hazards, evaluating the disaster resilience of mountainous villages is crucial for sustainable regional development. This study proposes an integrated resilience assessment framework specifically designed for high-altitude, tourism-dependent ethnic villages. The framework extends the classical DPSIR model by incorporating an Adaptability (A) dimension, which quantifies traditional ecological knowledge and community learning capacity. To address the class imbalance typical of small-sample geological hazard datasets, the study integrates the SMOTE oversampling technique with a multi-model evaluation chain (IVM-SVM-RF). This method overcomes the generalization limitations of machine learning models in small-sample environments. The results show: (1) Model Performance Breakthrough: The SMOTE-enhanced Random Forest (S-RF) model outperforms both SVM and IVM models, with the highest performance (AUC = 0.753, Kappa = 0.754). It is particularly effective in identifying low-resilience areas under small-sample conditions, confirming that SMOTE augmentation corrects class imbalance and improves model accuracy in capturing marginal low-resilience zones. (2) Spatial Differentiation: Zhangzha Town exhibits distinct “topography-constrained economic clustering” patterns of resilience. The high-resilience core zone (13.05%) is concentrated in the Ganhaizi sector, driven by socio-economic adaptability, while the extremely low-resilience zone (9.42%) is scattered along the southern periphery, influenced by steep terrain and delayed responses. (3) Nonlinear Drivers: Feature importance analysis identifies Fractional Vegetation Cover, Rainfall, and Distance from Roads as key resilience determinants. Additionally, “soft resilience” factors, such as Building Disaster Resistance and Villagers’ Disaster Awareness, play significant roles in mitigating physical risks.

## Introduction

 Global warming and the increasing frequency of extreme geological events have exposed mountain social–ecological systems (SESs) to growing compound risks^1^. Villages located along active fault zones and intensively developed for tourism are particularly vulnerable. While these villages play an important role in regional economic development, the combination of concentrated human activity and fragile natural conditions significantly increases disaster exposure, posing challenges to sustainable development^2^. In this context, there has been a gradual shift from conventional disaster prevention approaches toward resilience-oriented governance, which emphasizes the capacity of systems to absorb disturbances, recover, and adapt over time. This transition has become an important focus in international research on disaster risk reduction and sustainable development^3^.

Resilience assessments in the disaster literature generally follow two paradigms: outcome-based and capacity-based. Outcome-based resilience is measured through post-disaster loss data and recovery trajectories, requiring longitudinal observations that are rarely available in remote mountain regions. Capacity-based resilience, by contrast, evaluates the latent ability of a socio-ecological system to absorb and adapt to hazard impacts, using a set of environmental, socioeconomic, and institutional indicators as proxies. This study adopts the capacity-based paradigm. Accordingly, what we term “disaster resilience” throughout this paper therefore refers to inherent resilience capacity: the pre-existing configuration of physical and social conditions that jointly determine a community’s potential to withstand and recover from future hazard events.

Despite the growing application of resilience theory in urban systems, its direct transfer to mountain tourism villages remains challenging. One key issue concerns the conceptual distinction between susceptibility and resilience. Many existing studies focus primarily on natural hazard factors, such as topography and lithology, thereby emphasizing static susceptibility. In contrast, resilience reflects the adaptive capacity of communities, including cultural practices and institutional responses. Neglecting this social dimension limits the understanding of self-organizing mechanisms within coupled human–environment systems^6,11^.

Another challenge relates to the limitations of existing evaluation frameworks. The traditional DPSIR model is often applied as a linear causal chain linking driving forces, pressures, states, impacts, and responses. However, such a structure struggles to represent the nonlinear feedback dynamics inherent in complex socio-ecological systems. As Gari et al. observed in their review of DPSIR applications to coastal social-ecological systems, the framework has undergone numerous contextual adaptations where community agency plays a central role in system functioning^7,4^. For ethnic villages rich in traditional ecological knowledge, omitting the Adaptability (A) dimension precludes the quantification of a community’s long-term evolutionary and learning capacities. State (S) captures only the momentary condition of the system, while Response (R) is typically event-triggered and heavily reliant on external administrative coordination. Adaptability (A), in contrast, is a culturally embedded, endogenously sustained capacity that operates continuously without external instruction and does not fluctuate with short-term events. Without an explicit A dimension, the evaluation framework would misattribute such capacities as either transient conditions or externally driven interventions, thereby systematically underestimating the self-organizing resilience of these communities.

A further challenge arises from methodological constraints in spatial prediction. Machine learning models, such as Random Forest and Support Vector Machine, have demonstrated strong performance in nonlinear modeling. However, in geological hazard contexts, hazard samples are typically sparse and highly imbalanced compared with non-hazard samples. Without appropriate treatment, this imbalance can bias the model toward the majority class and increase omission errors, thereby affecting the reliability of resilience assessment.

In response to these challenges, this study develops a resilience assessment framework tailored to complex mountainous environments, using Zhangzha Town in Jiuzhaigou as a case study. The study extends the DPSIR framework by incorporating an Adaptability dimension, with a particular focus on quantifying traditional ecological knowledge and community-based adaptive practices in Tibetan and Qiang villages.

Methodologically, SMOTE is combined with a progressive multi-model chain (IVM–SVM–RF) to address prediction bias caused by extreme sample imbalance. This approach improves the identification of low-resilience areas in small-sample conditions. In addition, by examining the nonlinear drivers of spatial variation in resilience, the study provides a basis for more targeted zoning and resilience-oriented planning in high-altitude tourism regions.

## Research methods and data sources

### Study area overview

**Fig. 1:**
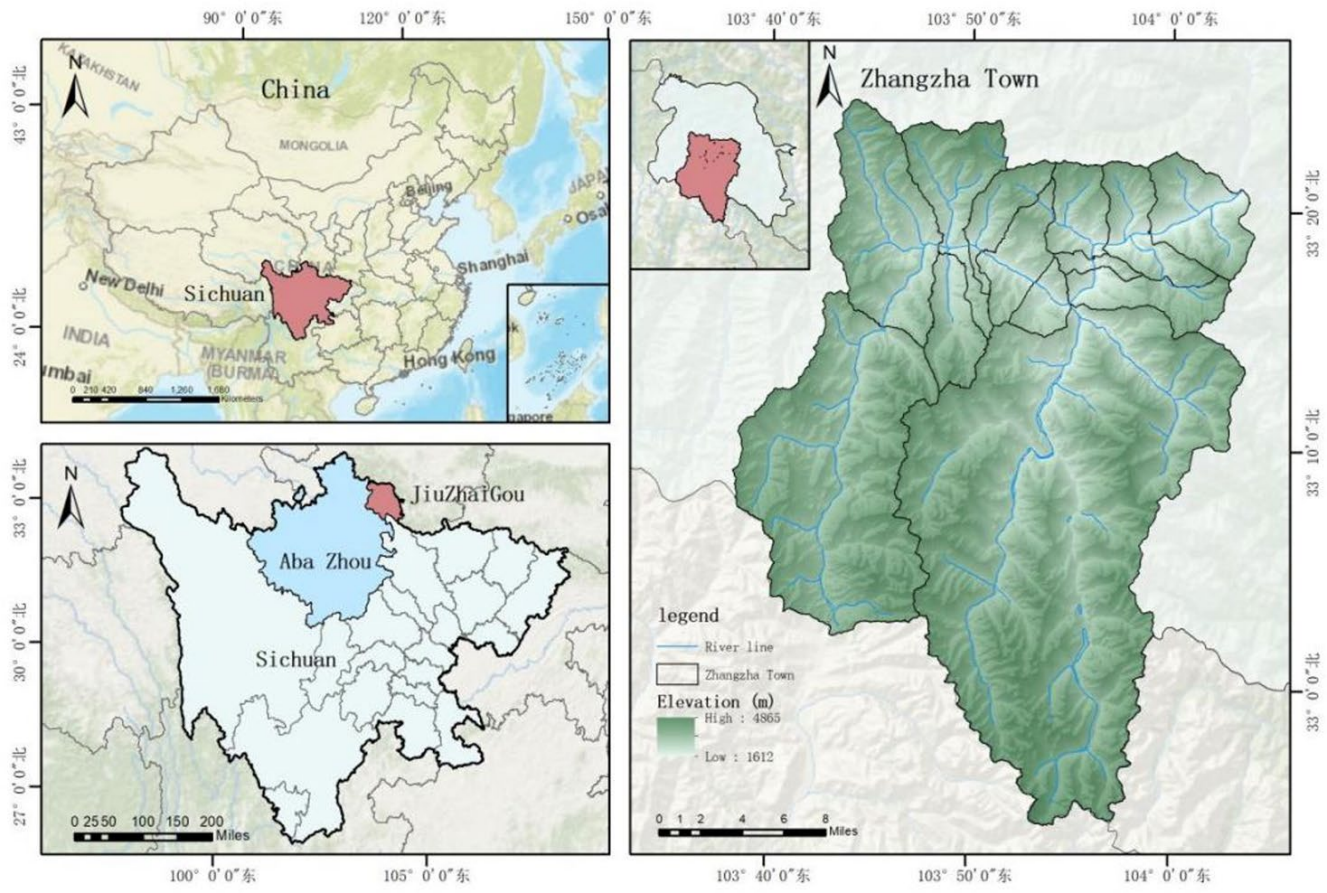
Geographic location of the study area (a), structural map of Jiuzhaigou County (b), spatial distribution of geological hazards in Zhangzha Town (c). Maps were generated using ArcGIS 10.8 (Esri Inc., USA; https://www.esri.com/en-us/arcgis/products/arcgis-desktop/overview).

Zhangzha Town is located in the transitional zone between the eastern margin of the Qinghai–Tibet Plateau and the Sichuan Basin (33°02′–33°21′ N, 103°38′30″–104°03′40″ E). The region features a typical high mountain–canyon landscape with complex geological structures, making it one of the most geologically sensitive areas in China. The significant elevation range (1,612–4,865 m) and intense relief contribute to inherently unstable slopes. After the 2017 Jiuzhaigou Ms 7.0 earthquake and subsequent aftershocks, tectonic stress was further released and redistributed, leading to frequent landslides, collapses, and other related hazards(Fig. 1).

As the main gateway to Jiuzhaigou, a UNESCO World Heritage site, Zhangzha Town displays a typical pattern of tourism-driven tension in human–environment interactions. On one hand, the dense concentration of homestays, restaurants, and transportation infrastructure along river valleys and fault zones has significantly increased population density and asset exposure^5^. On the other hand, local Tibetan and Qiang communities have developed substantial adaptive knowledge through long-term disaster response practices, including site selection strategies, construction techniques, and everyday preventive measures^35,39^. This strong coupling of human activities and natural processes makes Zhangzha Town an exemplary case for studying “social–ecological system resilience” in mountainous communities.

### Data sources and preprocessing

To comprehensively capture the natural environment, human activity intensity, and community resilience characteristics of the study area, this study integrates heterogeneous data from multiple sources, including remote sensing imagery, statistical yearbooks, field surveys, and government open data (Table 1). The data includes five key categories of indicators: geological hazards, topography, climate, socioeconomic conditions, and resilience responses.

To ensure consistency across model inputs, all spatial datasets were resampled to a unified 30 m × 30 m grid resolution using bilinear or nearest-neighbor interpolation, and normalized via min–max scaling to eliminate dimensional differences that could bias model training. For socioeconomic and survey-based indicators originally collected at the village administrative level, values were allocated to all grid cells falling within each village boundary, thereby maintaining a uniform spatial resolution when integrating multi-source data into the machine learning pipeline. While this spatial allocation introduces a degree of scale generalization, it enables soft sociocultural indicators and hard environmental variables to be fused within a single analytical framework.

## Research methodology

**Fig. 2:**
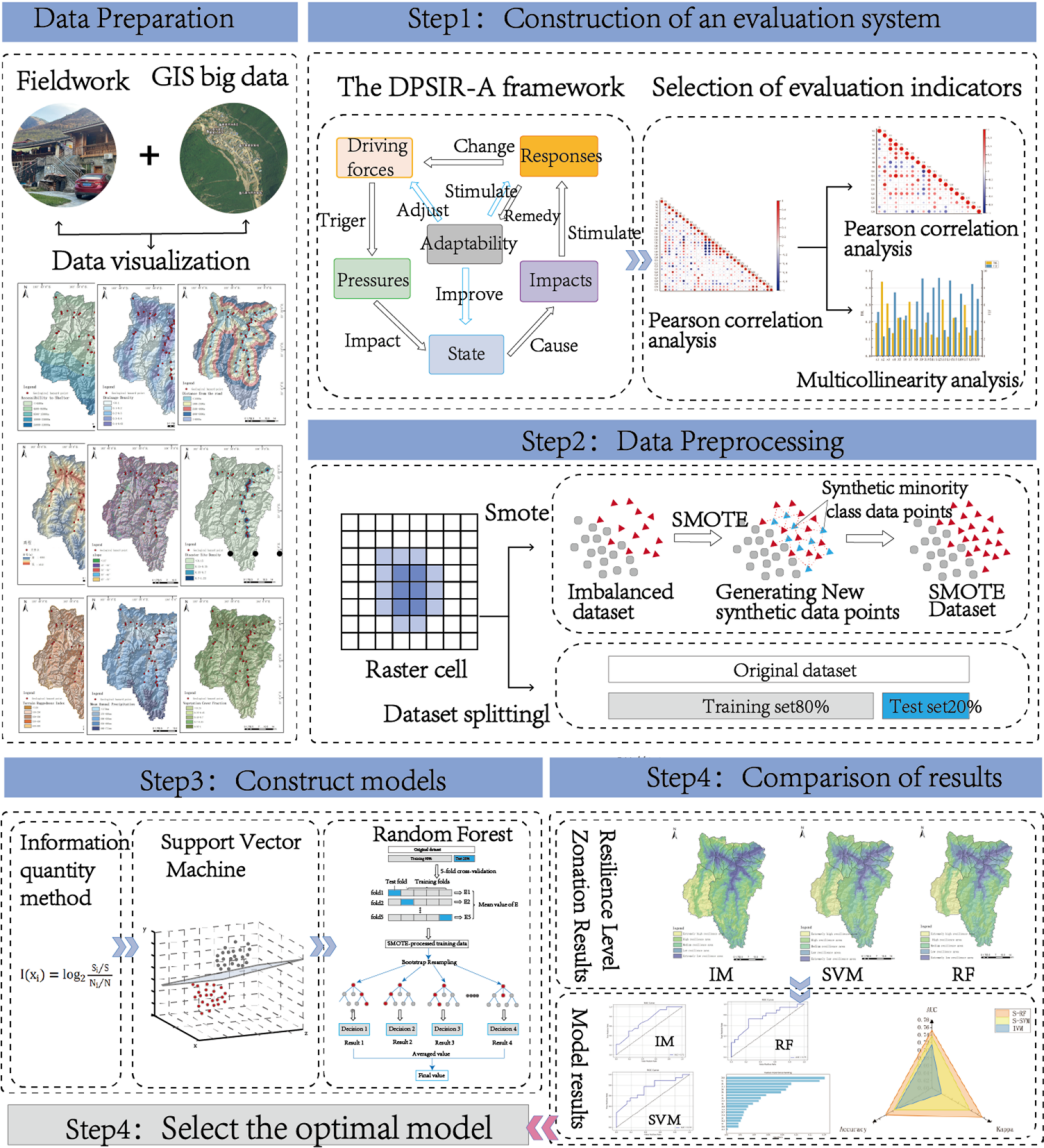
Study Framework Diagram.

This study develops an integrated technical framework of “theory-driven, data-enhanced, model comparison, and spatial analysis” to systematically identify resilience distribution within the social-ecological system of Zhangzha Town and its key influencing factors (Fig. 2).

1. Construction of the evaluation system.

Based on the enhanced DPSIR-A model, 19 core indicators were selected across six dimensions: Driving Force (D), Pressure (P), State (S), Impact (I), Response (R), and Adaptability (A). Expanding on the classical disaster-environment-society evaluation framework, the indicator system incorporates community-level adaptive behaviors and disaster prevention capacities, providing a theoretical basis for resilience modeling.

2. Data preprocessing and augmentation.

Using 69 historical disaster samples as positive class points, an equal number of non-disaster samples were randomly generated as negative class points. Given the small overall sample size, the SMOTE algorithm was applied to generate synthetic samples, expanding the training set, mitigating model bias from sample imbalance, and improving learning stability.

3. Multi-model comparison and modeling.

Three representative models—Information Value Model (IVM), Support Vector Machine (SVM), and Random Forest (RF)—were used for resilience zoning prediction. Model performance was evaluated using ROC curves, AUC values, and Kappa coefficients to identify the optimal model.

4. Identification of spatial heterogeneity and zoning.

Based on the top-performing model, key drivers were identified, and a map of resilience spatial differentiation was generated. By integrating topography, settlement patterns, and infrastructure layouts, resilience gradients and driving mechanisms across regions were analyzed, providing decision support for future risk governance and community resilience enhancement.

## Evaluation model construction

### Sample Balancing: SMOTE-based oversampling

Geological hazard samples typically exhibit significant imbalance, where the number of hazard points is far lower than that of non-hazard points. This imbalance causes model decision boundaries to lean toward the majority class, impairing the identification of low-resilience areas. To mitigate this issue, this study employs the SMOTE algorithm to augment minority class samples through interpolation. The core principle of SMOTE involves selecting a minority class sample$\:{x}_{i}$ and one of its k nearest neighbors$\:{\widehat{x}}_{i}$ in the feature space, then generating a new synthetic sample along the line segment connecting them:

1 $$\:{x}_{syn}={x}_{i}+\lambda\:\cdot\:\left({\widehat{x}}_{i}-{x}_{i}\right)$$

where$\:{\uplambda\:}$ ∈[0, 1] is a random number. Unlike simple duplication, SMOTE effectively expands the distribution range of the minority class in multidimensional space, avoiding overfitting. This study constructed an initial minority class dataset based on 69 hazard points. After generating balanced data via SMOTE, the test set F1-Score improved by 6.2%, significantly enhancing the model’s capacity to identify low-resilience regions^14,28^.

### Evaluation model comparison chain

#### Information Value Model (IVM) — Statistical Baseline

IVM quantifies the contribution of each factor by calculating differences in hazard occurrence probabilities across factor classes. The information value is calculated as follows:

2 $$\:I({x}_{i},H)={ln}\frac{{N}_{i}/N}{{S}_{i}/S}$$

Where $\:{N}_{i}$ represents the hazard area within *factor* i,$\:\:{\:S}_{i}$ denotes the total area of that factor, while *N and S* denote the total hazard area and total area of the study region, respectively. As a statistical baseline model, IVM clearly presents the linear correlation between factors and disasters, serving as a fundamental reference for subsequent models^19,11,42^.

## Support Vector Machine (SVM) — Classical machine learning

SVM performs classification by constructing an optimal hyperplane in a high-dimensional feature space. This study employs the Radial Basis Function (RBF) kernel to capture nonlinear relationships among indicators[^32^], enabling the model to handle complex interactions between geomorphology and human activities. Parameter tuning utilizes grid search and five-fold cross-validation to ensure model robustness^15,16,18^.

### Random Forest (RF) — Ensemble learning optimization

RF conducts classification through ensemble voting of multiple decision trees. It offers several advantages, including robustness to outliers and missing values, insensitivity to high-dimensional data, and stable performance under spatial heterogeneity. In addition, variable importance can be evaluated using out-of-bag (OOB) error^21–23^. In this study, the RF model is configured with 500 decision trees, and the Gini index is used to assess feature importance, enabling the identification of key driving factors.

### Construction of the disaster resilience evaluation system

#### Design of the DPSIR-A theoretical framework

To transcend the traditional “static exposure-risk” paradigm in disaster assessment, this study introduces the Adaptability (A) dimension into the classical DPSIR framework, forming the extended DPSIR-A model^4,12^.

In this framework, Adaptability (A) is defined as an inherent capacity of a village to mitigate hazard impacts over the long term through culturally transmitted knowledge, accumulated local experience, everyday practices, and community-based social organization. It manifests in the autonomous risk-avoidance behaviors of villagers: knowing which roads become hazardous after an earthquake, which slopes to avoid during the rainy season, and how to reinforce roof beams using traditional techniques. It differs from both State (S) and Response (R). State reflects the actual condition of physical infrastructure and institutional arrangements at a given moment, while Response refers specifically to official emergency actions that are triggered after a disaster and coordinated through external administrative channels.

Adaptability has two defining characteristics: it is temporally stable, and it is endogenously maintained by the community itself. These qualities allow it to function as a persistent buffer that moderates the entire D-P-S-I-R chain. The inclusion of Adaptability as a separate dimension thus captures a form of resilience that neither State nor Response can adequately measure, filling a conceptual gap in the original framework.

##### The D-P-S-I-R chain

This chain illustrates how tourism development (D) transforms into terrain pressure (P), alters environmental states (S), generates socio-economic impacts (I), and ultimately triggers policy responses (R).

##### The feedback effect of Adaptability (A)

**Fig. 3:**
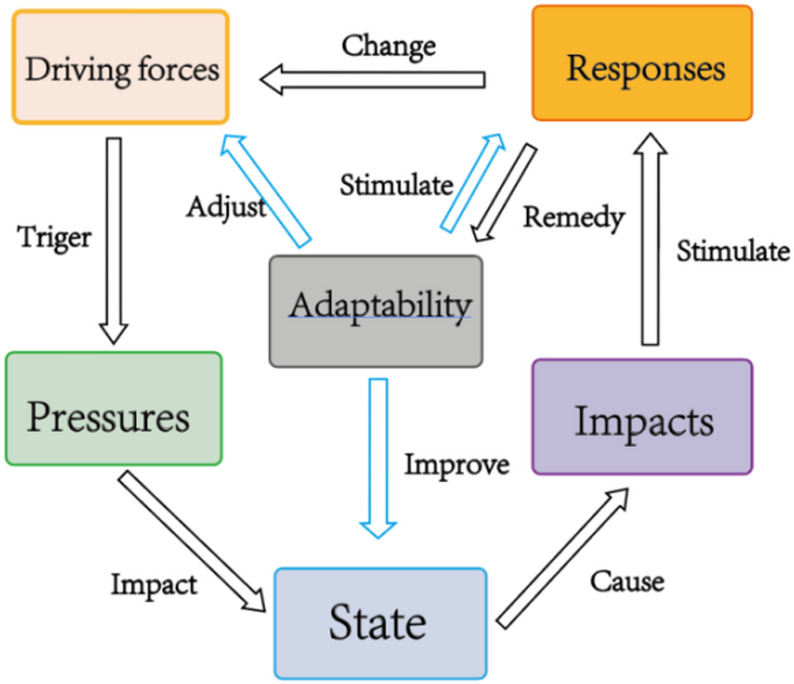
Schematic of the DPSIR-A Model Framework.

Adaptability not only directly mitigates pressure (P) but also optimizes system state (S) by enhancing the efficiency of response (R). For example, traditional Tibetan and Qiang construction wisdom (A) significantly enhances the disaster resilience of buildings (S) without altering terrain conditions (P). (Fig. 3)

### Indicator selection and statistical testing

To ensure the independence and scientific rigor of the evaluation system, this study employs a two-step approach of “qualitative preliminary selection followed by quantitative refinement”:

#### Initial Indicator Pool

Combining literature review and field research in Zhangzha Town, an initial indicator database comprising 31 factors was constructed[^26^]. Indicator selection referenced geographical-constructional characteristics of villages in earthquake-prone areas^27,33^ and hazard resilience research findings^26,36^.

#### Redundancy Removal

**Fig. 4:**
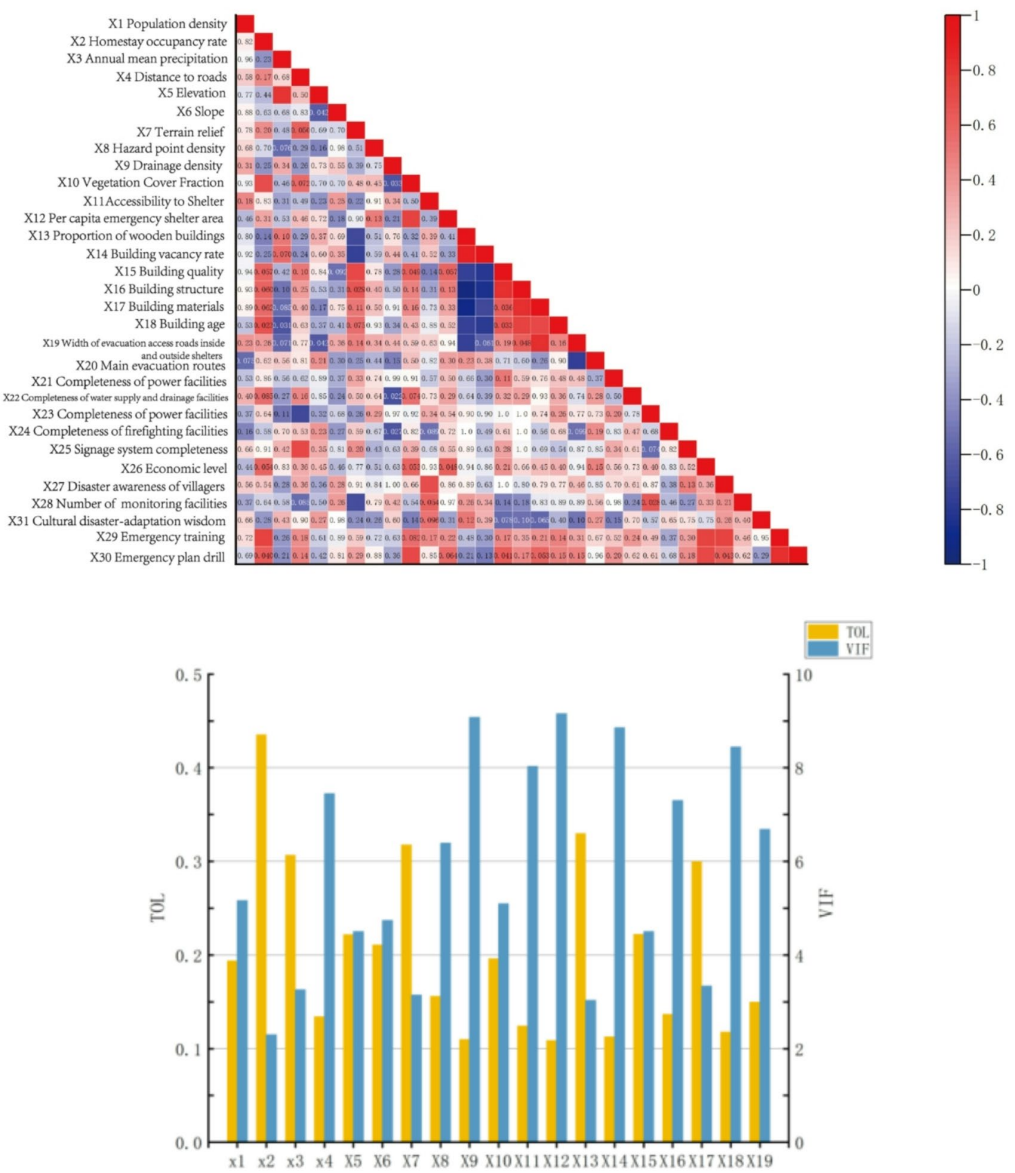
Indicator Correlation Heatmap and Collinearity Test Results.

Pearson correlation analysis was applied to eliminate redundant factors ($\:\left|\mathrm{r}\right|\ge\:0.8$), such as the high correlation between “building vacancy rate” and “number of homestays.“(Fig. 4).

**Multicollinearity Diagnosis (**VIF): The remaining factors underwent collinearity testing. Results showed that the variance inflation factor of the final 19 retained indicators was$\:\:\left(\mathrm{V}\mathrm{I}\mathrm{F}<10\right)$, with a tolerance of$\:\left(\mathrm{T}\mathrm{O}\mathrm{L}>0.1\right).\:$This indicates no significant multicollinearity among the indicators, meeting the input requirements for machine learning models.

During the feature selection phase, this study not only relied on Pearson correlation coefficients to eliminate highly correlated factors with |r| > 0.8 to reduce data redundancy but also incorporated multicollinearity diagnostics.

Experimental results show that the variance inflation factors of the 19 retained indicators range from 1.12 to 3.45, well below the critical threshold of 10, while their tolerances all exceed 0.2(Fig. 4). These results indicate that the evaluation dimensions are statistically independent, ensuring that subsequent machine learning models will not experience weight distortion due to variable collinearity during Recursive Feature Elimination (RFE). Consequently, this enhances the interpretability of disaster resilience assessment results.

### Disaster resilience evaluation indicator system

The final indicator system encompasses 6 criterion layers and 19 indicator layers, integrating both quantitative and qualitative representation (**Table **2). To ensure scientific rigor and independence, a two-step quantitative screening method was adopted to optimize the indicator set.

### Data standardization and grading rules

Given the varying dimensions of the indicators, this study uniformly standardized them into five grades (1–5), ranging from low resilience to high resilience.

**Fig. 5:**
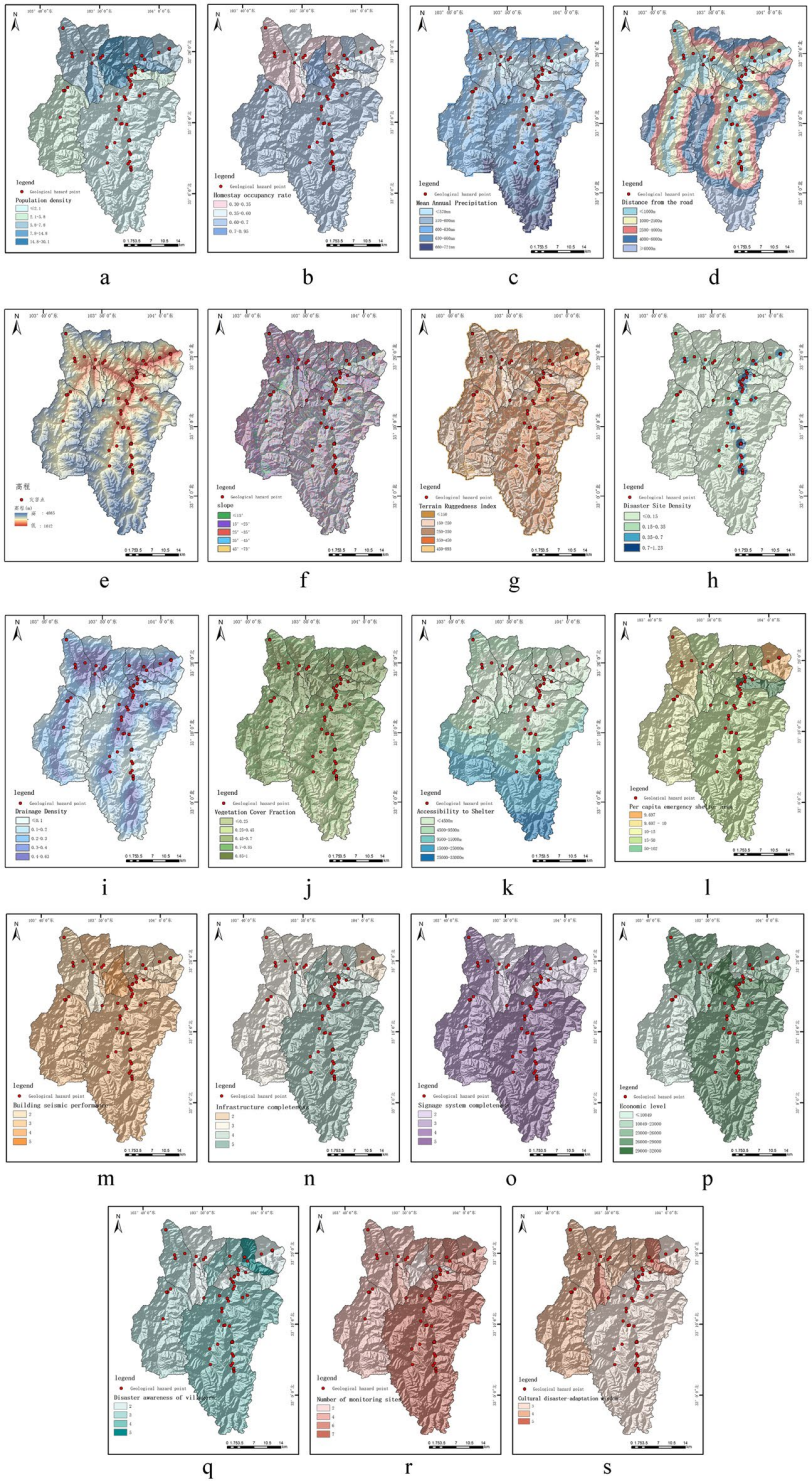
Spatial distribution matrix of disaster resilience evaluation indicators for Zhangzha Town (a–s represent 19 sub-indicators).

(1) For continuous quantitative indicators, which include both spatial baseline data and socioeconomic data, two grading approaches were applied according to the nature of the variable. Spatial baseline factors characterized by significant nonuniformity, such as elevation, slope, and fractional vegetation cover, were classified using the GIS Natural Breaks method. This technique identifies structural inflection points within the data, minimizing intra-class variation while maximizing inter-class variation, and thereby objectively reflects the nonlinear constraints that geographic elements impose on resilience. For socioeconomic indicators such as population density, homestay occupancy rates, and per capita income, the quantile method was applied. This approach effectively handles skewed statistical distributions and incorporates seasonal adjustments based on tourism industry standards, ensuring that the classification outcomes align with the tourism-driven dynamics of human-land relations in the study area.

 (2) For the quantification of qualitative and discrete indicators such as building disaster resistance performance and villagers’ disaster awareness, an ordered Likert scale was adopted. Field interview records and participatory mapping data were compiled, after which five experts in geology and rural planning conducted double-blind scoring. A unified correspondence rule linking feature descriptions to numerical values was established, and the scoring results were cross-validated to ensure consistency. This procedure safeguards the repeatability and objectivity of converting qualitative judgments into quantitative inputs. Among these indicators, the newly introduced Adaptability (A) dimension, termed Adaptive Disaster Prevention Intelligence, assesses a community’s capacity to integrate traditional disaster prevention wisdom with modern technology and management systems. Scoring is based on three sub-dimensions: technological integration, organizational coordination, and spatial protection. Five levels are defined to evaluate building construction, disaster prevention collaboration, and ecological space preservation. The complete scoring rubric is provided in the Supplementary Materials (Table S1) Fig. 5.

It should be noted that the standardized indicator values derived from the above procedures were not aggregated into a single composite resilience index prior to modeling. All 19 indicators were retained as independent feature dimensions and fed directly into the machine learning pipeline, allowing the SVM and RF models to learn nonlinear interactions and threshold effects that a fixed linear combination would fail to capture. The explicit linear combination of indicators was applied only within the Information Value Model, where it served as a transparent statistical baseline.

### Data preprocessing and model construction

#### Sample generation and balancing strategy

The generalization capability of machine learning models heavily depends on sample quality. This study constructed a balanced dataset comprising 138 observation points:

##### (1) Label definition and negative samples

69 field-verified historical geological hazard sites were designated as low-resilience samples (Label = 0), reflecting functional loss under disaster impacts. A conceptual note on the labeling logic is necessary. In this study, the occurrence of a geological hazard is treated as an observable indicator of functional failure under external stress, signaling that the local socio-ecological system was unable to maintain stability when exposed to a given set of physical pressures. Conversely, non-hazard points sampled under controlled conditions are not interpreted as inherently safe locations. Rather, their hazard-free status, recorded under comparable levels of tourism-driven exposure and after excluding unbuildable terrain, is understood to reflect a favorable configuration of internal adaptive and response capacities that effectively counterbalance physical pressures. Under this logic, the binary labels capture not the absence of hazard potential but the spatial manifestation of latent resilience capacity. The machine learning models are subsequently trained to distinguish the multivariate indicator profiles associated with functional failure from those associated with functional stability.

##### (2) Constrained random sampling (Absence)

**Fig. 6:**
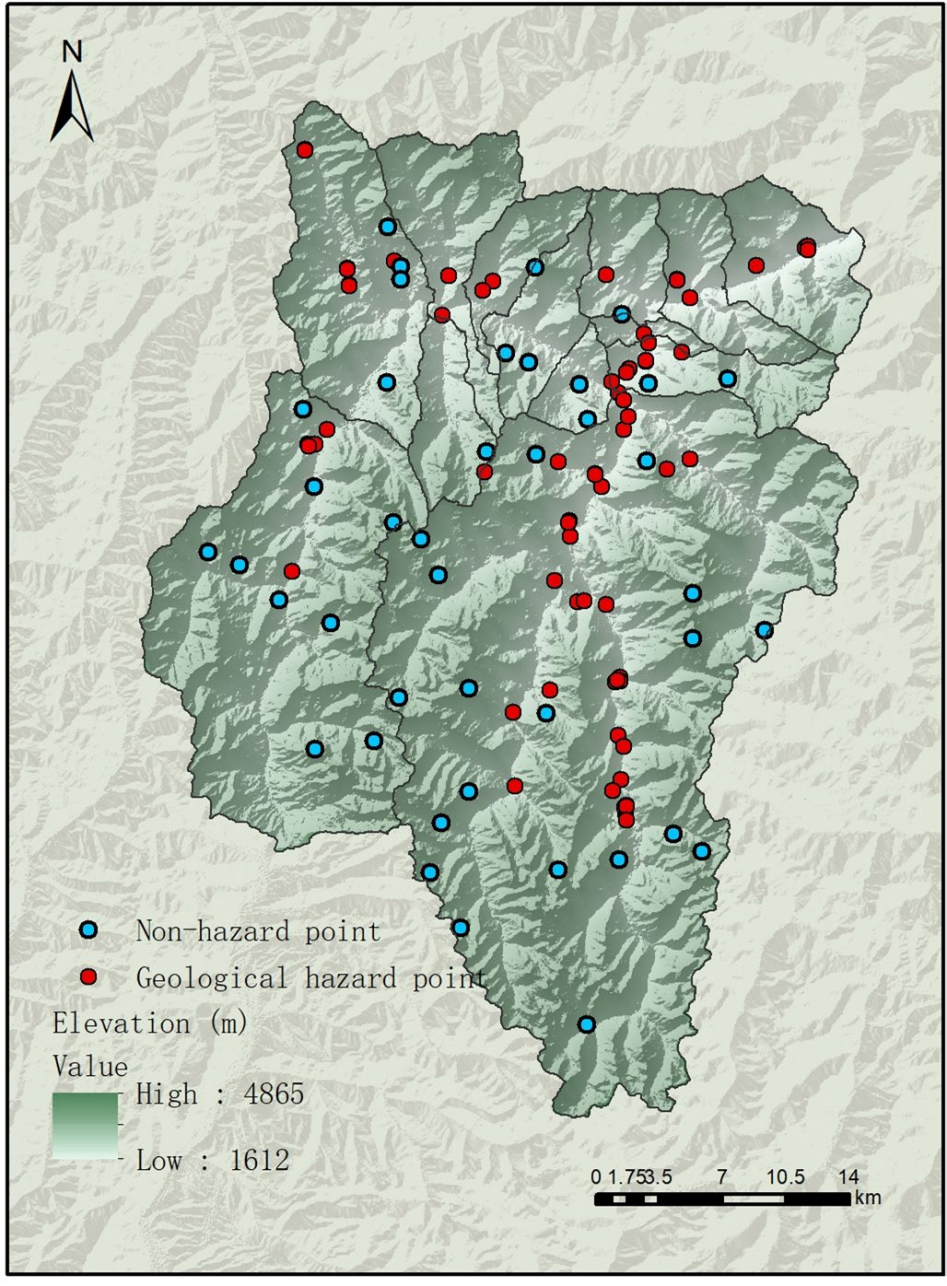
Spatial distribution of training samples.

To scientifically extract “high resilience” samples (Label = 1), constrained random sampling was applied in ArcGIS. A 500 m spatial autocorrelation buffer was established, and rivers, cliffs, and other unbuildable terrain were excluded, ensuring that all samples were confined to areas within a reasonable range of human activity. This procedure generated 69 non-hazard points (Fig. 6) and guarantees that both the observed samples and the synthetic samples produced by the SMOTE algorithm possess spatial plausibility.

##### (3) Multidimensional feature extraction

The “Extract Multi-Values to Point” tool was used to map 19 indicator attributes to 138 samples.

##### (4)Data quality control and augmentation

Continuous variables were Z-score standardized to eliminate dimensional effects.

**(5) SMOTE augmentation**: To address potential minority class sample scarcity in the five-level resilience classification, the SMOTE algorithm was applied. By searching for K = 5 nearest neighbors in the feature space and performing linear interpolation, synthetic samples were generated to balance the distribution weights across resilience levels and prevent model bias to the majority class. During SMOTE oversampling, the sampling ratio was determined using inter-sample distance evaluation, and the class proportions were adjusted to 1:1. Unlike simple random oversampling, SMOTE avoids mechanical duplication of observations. By selecting $k = 5$ nearest neighbors, this configuration effectively fills the gaps of “low resilience” features in the multidimensional attribute space while preserving the topological characteristics of the minority class. This preprocessing step significantly enhances the model’s accuracy in identifying marginal samples, boosting the F1-Score on the test set by approximately 6.2%. It addresses the issue of classification decision boundaries easily shifting toward the majority class in small-sample environments.

### Multi-model evaluation pipeline

The W-IVM model uses an explicit linear weighted combination. For each indicator, the information value of each class is first calculated using Eq. (2). These class-level information values are then summed with the AHP–Entropy composite weights reported in Table 3, yielding the total information value for each grid cell:

**Table 1:** Summary of Research Data Classification and Sources.

Data Category	Specific Indicators	Resolution/Scale	Data Source
Geological Hazards	Historical landslide, rockfall, and debris flow sites (69)	—	Geospatial Remote Sensing Ecological Network, Ministry of Natural Resources field surveys
Terrain Baseline	Elevation, slope gradient, aspect, terrain undulation	30 m	Geospatial Data Cloud (GDEMV3)
Environmental Resources	Vegetation Cover (NDVI), Annual Precipitation, River Network	30 m/1 km	NASA MODIS data, China Meteorological Data Network
Socioeconomic	Population density, per capita income, homestay occupancy rate	Village-level scale	County Statistical Yearbook, Government Statistical Bulletin
Resilience Response	Distribution of shelter spaces, building structures, disaster preparedness awareness	Field research	2023–2024 Field Measurements and Participatory Interviews

**Table 2:** Evaluation Framework for Disaster Resilience in Villages of Disaster-Prone Regions.

Criterion Level	Indicator Level	Definition and Scientific Definition	Attribute
Driving Force D	Population Density	Total number of permanent and transient residents per unit area	-
	Homestay Occupancy Rate	Reflects the concentration of people during peak tourist seasons	-
	Rainfall	Higher precipitation correlates with increased frequency of natural disasters	-
	Distance from Roads	The greater the distance, the poorer the transportation accessibility	-
Pressure P	Elevation	High-altitude areas carry elevated disaster risks	-
	Slope	Steep slopes prone to landslides, rockfalls, and other geological hazards	-
	Relief	The greater the terrain undulation, the higher the risk of geological hazards	-
	Hazard Point Density	(Number of hazard points/Total village area) × 100%	-
	Drainage Density	Total river length per unit area	-
	Fractional Vegetation Cover	(Forest area/Total village area) × 100%	+
Status S	Accessibility to Shelter	Distance for residents to reach the nearest shelter	-
	Emergency Shelter Area Per Capita	Effective shelter area/Permanent population	+
	Building Disaster Resistance Performance	Classification by Structural Type and Reinforcement Measures: Tibetan stone-and-wood structures (Grade 1), brick-concrete structures (Grade 3), and light steel (Grade 5) are optimal	+
	Infrastructure Completeness	Integrity rate of water supply/power/communication systems Incomplete (Level 1), Relatively Complete (Level 3), Highly Complete (Level 5)	+
	Signage System Completeness	Grading of completeness and clarity in signage for disaster warnings, evacuation routes, and shelters Incomplete (Level 1), Fairly Complete (Level 3), Very Complete (Level 5)	+
Impact I	Economic Level	Economic indicators such as per capita income and industrial development level	+
**Response** R	Disaster Awareness of Villagers	Villagers’ level of disaster awareness and willingness to participate in disaster prevention activities No awareness (Level 1), Moderate awareness (Level 3), Strong awareness (Level 5)	+
	Number of Geological Hazard Monitoring Stations	Number of observation stations within the area used for real-time monitoring of geological hazards such as landslides and debris flows	+
Adaptability A	Adaptive Disaster Prevention Intelligence	Integration level between traditional ecological engineering methods and modern technologies	+

Note: (+) indicates that this indicator enhances system resilience; (-) indicates that this indicator increases system vulnerability and reduces resilience.

**Table 3:** Comprehensive Weighting of Disaster-Resilient Evaluation Indicators Based on AHP-Entropy Weighting Method.

Objective Layer	Criterion Layer - (Weight)	Indicator Layer	AHP Weight	Entropy Weight Method Weight	Comprehensive Weight
Resilience Evaluation System for Geohazard-Prone Villages	Driving Force D (0.2252)	Population Density	0.032	0.081	0.05769
		Homestay Occupancy Rate	0.057	0.133	0.168729
		Rainfall	0.092	0.030	0.061429
		Distance from Roads	0.044	0.035	0.034276
	Pressure P (0.2748)	Elevation	0.048	0.133	0.142088
		Slope	0.064	0.023	0.032762
		Relief	0.021	0.133	0.062163
		Hazard Point Density	0.036	0.052	0.041665
		Drainage Density	0.015	0.081	0.027042
		Fractional Vegetation Cover	0.091	0.037	0.074939
	Status S (0.199)	Accessibility to Shelter	0.031	0.037	0.025529
		Emergency Shelter Area Per Capita	0.046	0.035	0.035834
		Building Disaster Resistance Performance	0.078	0.016	0.027777
		Infrastructure Completeness	0.015	0.034	0.011351
		Signage System Completeness	0.029	0.030	0.019363
	Impact I (0.0696)	Economic Level	0.070	0.028	0.043623
	Response R (0.1514)	Disaster Awareness of Villagers	0.089	0.023	0.04556
		Number of Geological Hazard Monitoring Stations	0.062	0.042	0.057957
	Adaptability A (0.08)	Adaptive Disaster Prevention Intelligence	0.080	0.017	0.030269

**Table 4:** Optimal Parameters for Random Forest Model.

Hyperparameter	Grid Search Initial Value	Optimal Value
n_estimators	(97, 100, 1)	99
max_depth	(5, 10, 1)	9
min_samples_split	(6, 8, 1)	6
min_samples_leaf	(1, 3, 1)	1

**Table 5:** Comparative Statistics of Resilience Grade Areas for Three Models (km²).

Model	High Resilience	Moderately High Resilience	Moderate Resilience	Lower Resilience	Low Resilience
**W-IVM**	159.85	425.23	384.85	244.85	133.77
**S-SVM**	182.06	371.95	385.06	250.41	137.84
**S = RF**	175.93	413.5	386.97	244.52	126.9

$$\:{I}_{total}=\sum\:_{j=1}^{19}{w}_{j}\bullet\:I({x}_{ij},H)$$

where w_j_ is the composite weight of indicator jj, and I(x_ij_,H)I(x_ij_,H) is the information value of the class to which grid cell ii belongs for indicator jj. This is a fully transparent, manually specified linear formula.

The S-SVM and S-RF models, by contrast, do not rely on any pre-specified combination formula. Both models receive the 19 indicators as independent features, with the labeled samples (disaster point = 0, non-disaster point = 1) serving as the supervision signal. Through optimization of their respective loss functions, they autonomously learn the mapping from the 19-dimensional feature space to the binary resilience label. This learned mapping is implicit and nonlinear—it cannot be reduced to a single weighted-sum equation—but it is precisely this characteristic that enables SVM and RF to capture interaction effects and threshold behaviors that the linear IVM baseline cannot represent.

All three models operate on identically preprocessed data. The performance differences reported in Table 6 therefore reflect the gap between manual linear combination and data-driven nonlinear learning in resilience identification—not any difference in input data or preprocessing.

**Table 6:** Performance Metrics Comparison of Three Models.

Model	AUC Value	Kappa Coefficient	Precision
W-IVM	0.7142	0.6290	0.7108
S-SVM	0.7368	0.7160	0.7154
S-RF	0.7526	0.7540	0.7410

### Statistical baseline: Weighted information vector model (W-IVM)

The IVM quantifies the contribution of each factor level to resilience. This study introduces a combined weight based on AHP and the Entropy Weight Method (**Table 3**) to refine the traditional IVM. Through weighted overlay analysis using a raster calculator, results indicate that the IVM can preliminarily identify extremely low-resilience areas; however, its linear assumption overlooks the complex interactions within socio-ecological systems.

### Support Vector Machine (S-SVM)

An S-SVM model was constructed using Python, employing a Radial Basis Function (RBF) kernel to map 19-dimensional features into high-dimensional space. The optimal hyperplane was identified through iterative search of the regularization parameter c and kernel parameter g. The SMOTE-augmented dataset significantly enhances the classification margin in the feature space, improving the model’s ability to recognize the “high pressure-low response” characteristics in the central flat terrain region.

### Random forest ensemble learning (S-RF)

To address the complex heterogeneity of mountainous environments, a Random Forest model optimized via grid search was constructed. To mitigate overfitting risks caused by sparse geological hazard samples, five-fold cross-validation was implemented during model training[^24^]. The 300 SMOTE-enhanced samples were evenly divided into five folds, with four folds sequentially used as the training set and one fold as the validation set. After multiple iterations, the optimal hyperparameter combination is shown in **Table **4. This approach maximizes the model’s generalization capability under limited samples, ensuring its accuracy in capturing the nonlinear evolution characteristics of disaster resilience. To achieve optimal predictive performance, exhaustive grid search cross-validation was employed to explore the hyperparameter space of the random forest. Key optimizations focused on the number of decision trees and the maximum number of features. Experiments revealed that the out-of-bag (OOB) error stabilized when the number of trees reached 99. Furthermore, setting min_samples_leaf to 1 balanced model bias and variance, preventing overfitting caused by excessive tree depth. This data-driven parameter configuration scheme improved the model’s Kappa coefficient by 0.038 compared to default settings. This approach effectively captured the complex interaction between relief and accessibility to shelter.

**Fig. 7:**
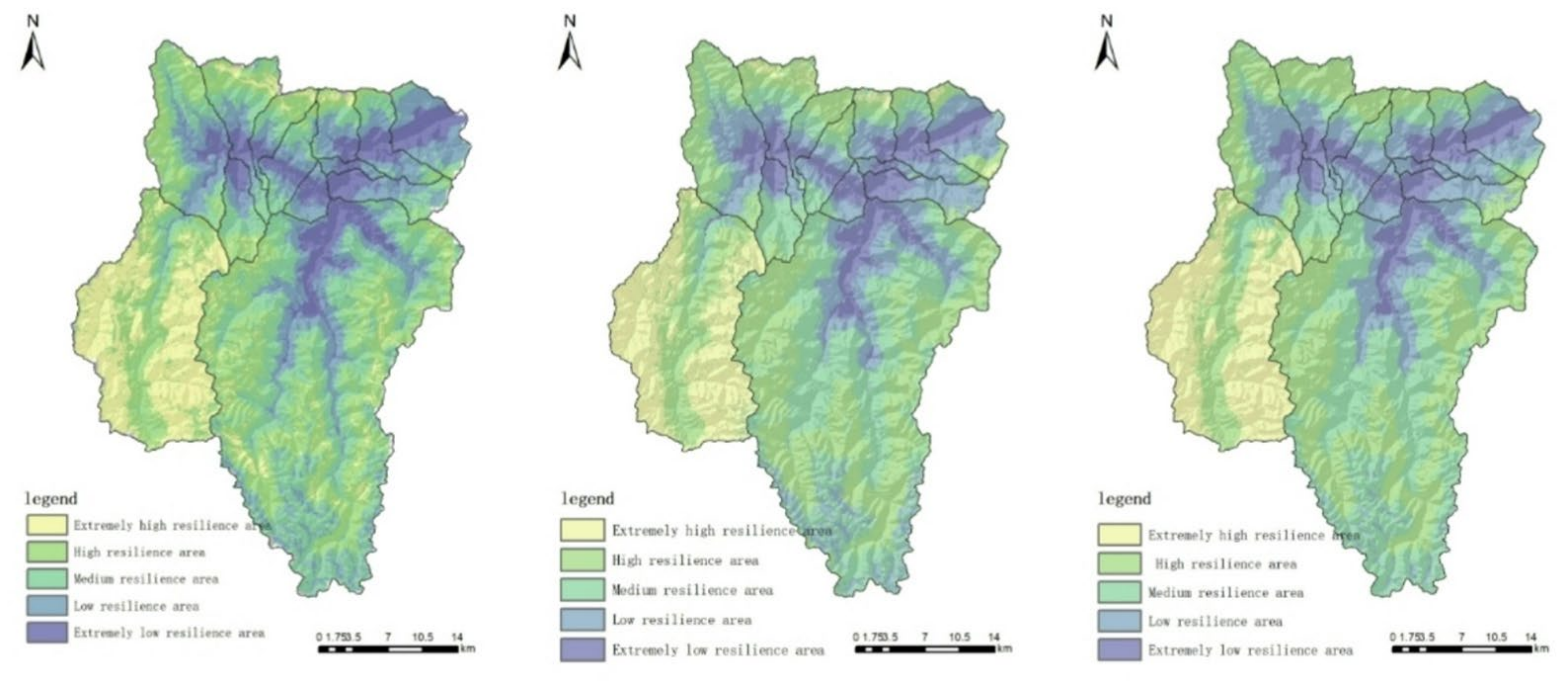
Spatial Distribution Comparison of Multi-Model Evaluation Results (Panel Map: IVM, SVM, RF).

#### Spatial distribution comparison of model zoning results

The three trained models were applied to the entire grid cells of Zhangzha Town. Using the Natural Breaks method, the results were divided into five resilience levels (Fig. 7).

Consistency Analysis: All three models identified areas of extremely low resilience that highly coincide with historical hazard points, primarily distributed in southern canyons and central human activity clusters.

Differential Evaluation: The S-RF model delineates more distinct resilience thresholds (**Table **5) and demonstrates stronger spatial continuity in identifying high-resilience zones. It captures subtle nonlinear interactions between relief and accessibility to shelter.

### Research findings and analysis (Results)

#### Model performance comparison and accuracy validation

Model performance was evaluated using Accuracy, the Kappa coefficient, and the Area Under the Curve (AUC) (**Table **6).Among the three models, the S-RF model achieved the best overall performance, followed by the S-SVM model and the IVM. Specifically, the S-RF model reached an AUC of 0.753 and a Kappa value of 0.754, outperforming S-SVM (AUC = 0.737, Kappa = 0.712) and IVM (AUC = 0.714, Kappa = 0.685).

**Fig. 8:**
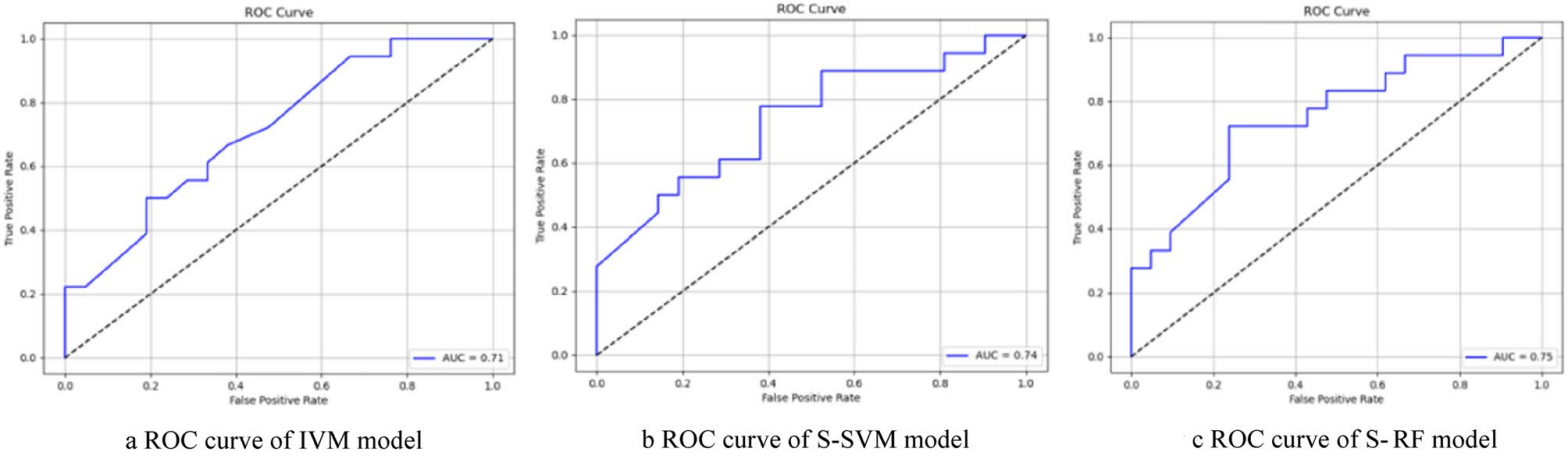
Comparison of ROC Curves for Three Models.

Although the absolute AUC values are moderate, they are comparable to or exceed those reported in other small-sample geological hazard susceptibility studies in complex mountainous regions (typically 0.70–0.80)^8–10,14^. The use of SMOTE further improved model performance, with an increase of approximately 6.2% in F1-score on the test set. This improvement indicates a stronger ability to identify low-resilience minority samples and confirms the effectiveness of SMOTE in addressing class imbalance. (Fig. 8).

#### Spatial differentiation and village-scale analysis of disaster resilience

**Fig. 9:**
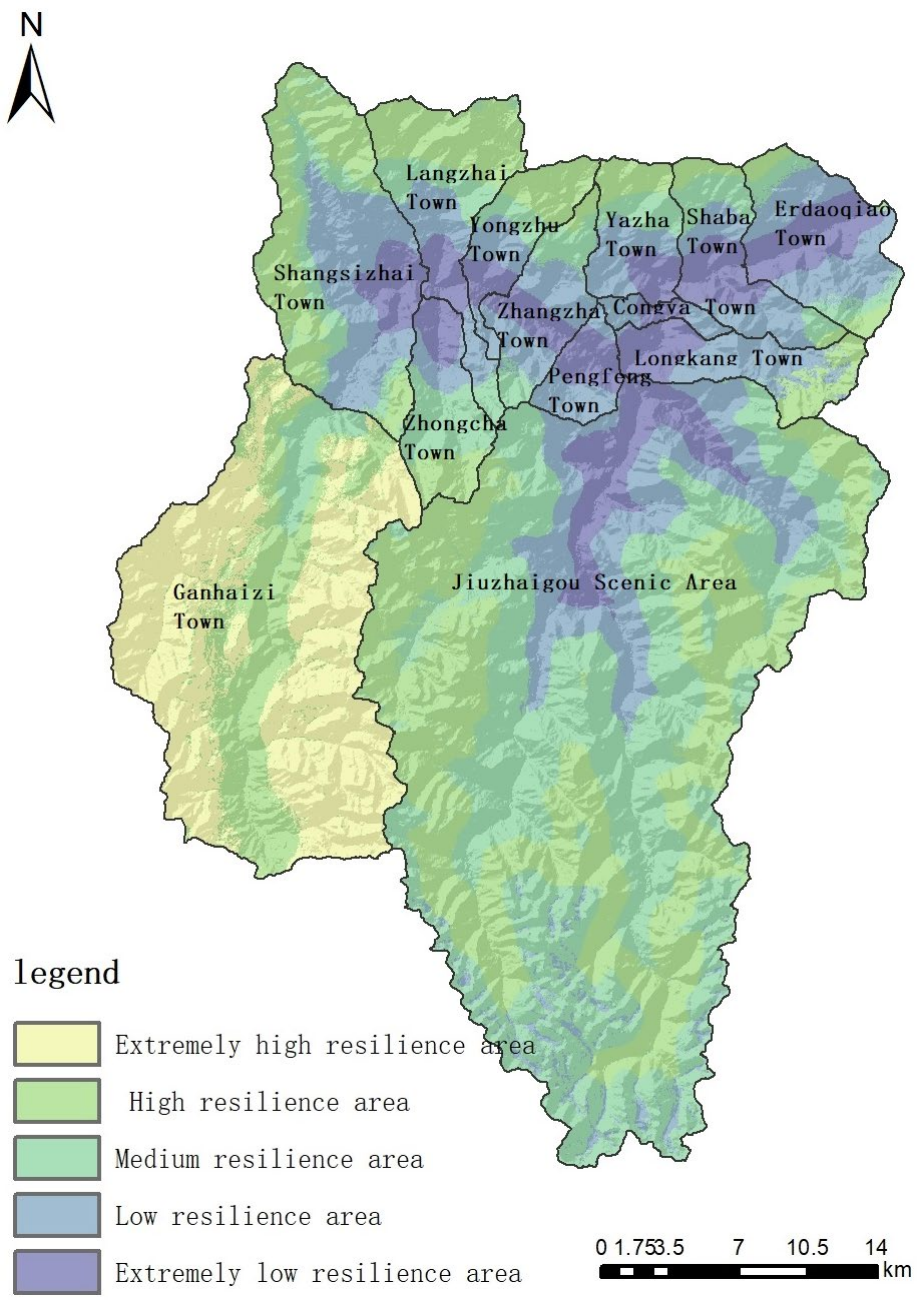
Final Resilience Classification Map for Zhangzha Town.

The spatial distribution derived from the optimal S-RF model shows clear heterogeneity across Zhangzha Town, characterized by strong topographic sensitivity and evident economic clustering (Fig. 9).

High-resilience areas are mainly concentrated around the Ganhaizi Community, forming a clustered pattern. These areas are generally located at moderate elevations with relatively gentle slopes and benefit from their role as administrative and tourism centers. The combination of stronger socio-economic adaptability and more developed infrastructure contributes to their higher resilience levels.

In contrast, extremely low-resilience zones are primarily distributed along the southern margins and steep canyon areas, including the edges of Shangsi Village. These areas account for nearly half (49.28%) of the recorded hazard points and exhibit very limited accessibility to shelters, making them critical targets for disaster prevention and risk management.

**Fig. 10:**
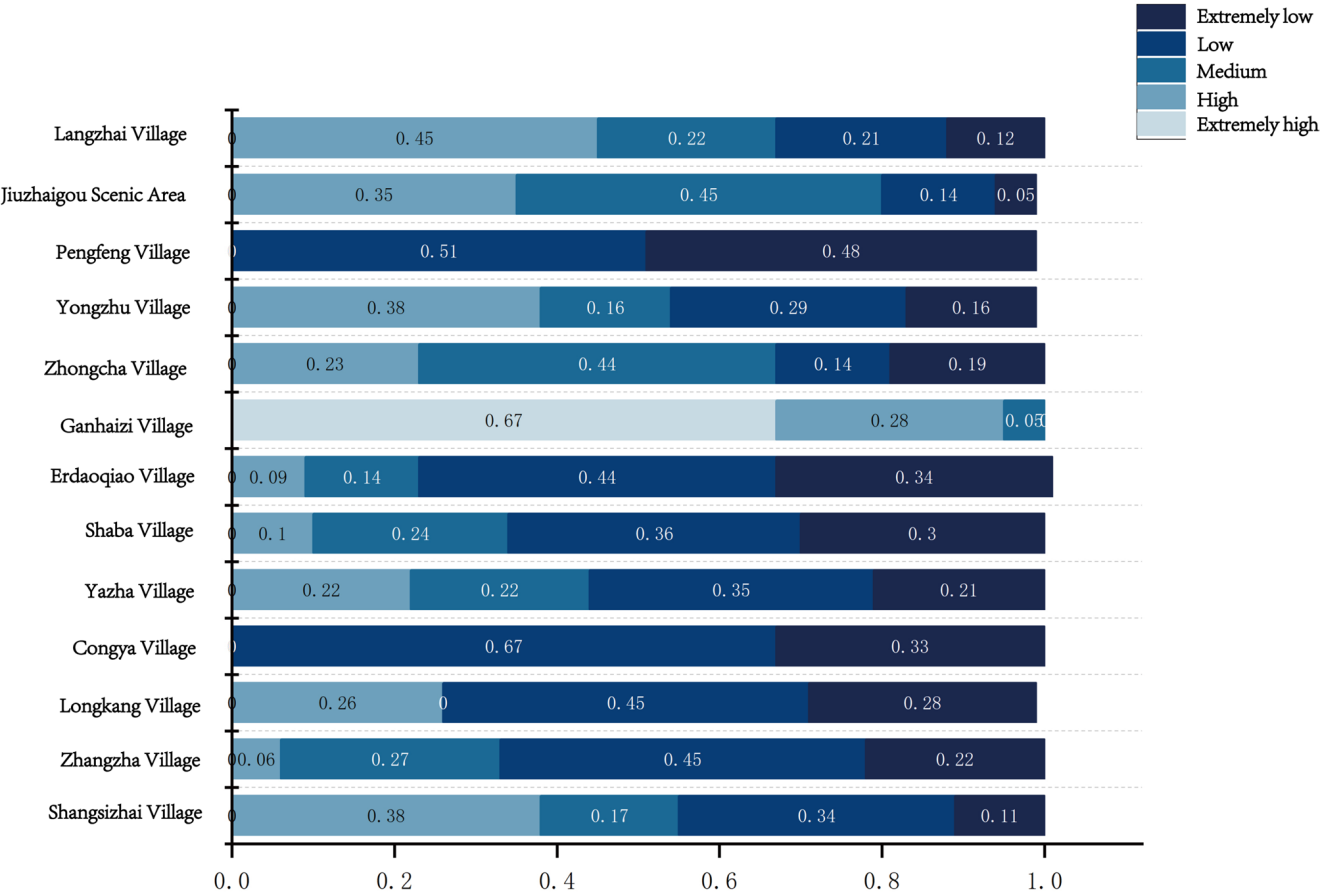
Overlay Bar Chart of Resilience Level Composition by Village.

At the village scale, notable variability can be observed. Ganhaizi Village and Congya Village are characterized by relatively high resilience, whereas Erdaoqiao Village shows a pronounced imbalance, with low-resilience areas covering approximately 68% of its total area. This pattern reflects a combination of high environmental pressure and insufficient response capacity (Fig. 10).

#### Identification of key drivers and contribution analysis

**Fig. 11:**
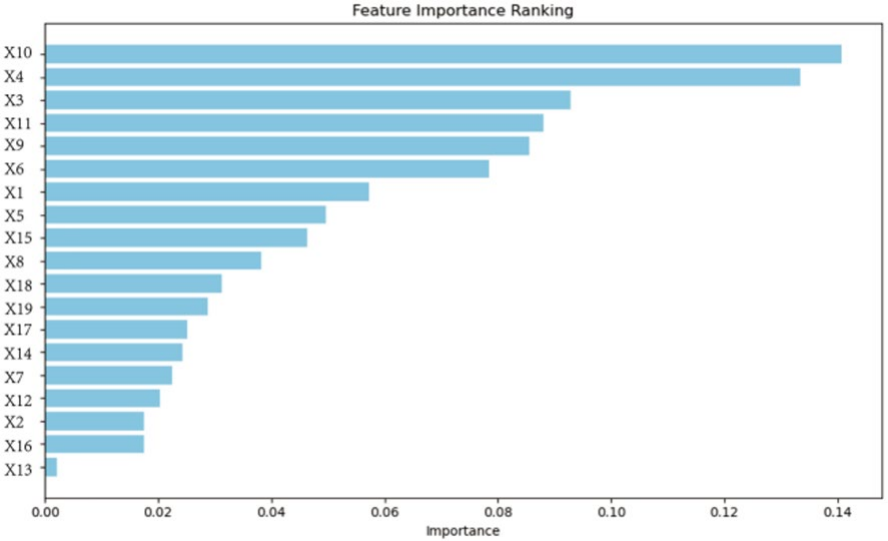
Feature importance ranking diagram of the S-RF model.

The feature importance ranking obtained from the S-RF model (Fig. 11) reveals a clear hierarchical structure among the factors influencing disaster resilience. Natural environmental variables, particularly relief and annual precipitation, remain the dominant drivers, as they determine the baseline vulnerability of the system.

Following these natural factors, variables associated with the Adaptability dimension, such as building disaster resistance and villagers’ disaster awareness, show high importance. This finding highlights the critical role of social adaptive capacity in mitigating environmental pressures. In high-risk areas such as Zhangzha Town, long-term accumulated knowledge of earthquake-resistant construction and strong community-based disaster awareness provide essential support for maintaining system functionality under extreme conditions. This result further supports the explanatory value of the DPSIR-A framework in capturing the interaction between human and environmental systems.

Another notable result is the role of distance from roads, which ranks among the top contributing factors. Roads exhibit a dual effect: on the one hand, slope cutting and terrain disturbance associated with road construction can increase exposure to hazards[^17^]; on the other hand, roads serve as key infrastructure for evacuation and emergency response, enhancing resilience. This duality reflects a typical trade-off in mountainous infrastructure systems.

In addition to identifying the relative importance of different factors, the S-RF model also captures nonlinear relationships among resilience drivers. For example, the influence of distance from roads is not only related to physical fragmentation, but also reflects a sensitivity threshold within approximately 500 m. Such nonlinear effects are difficult to detect using traditional linear models, highlighting the advantage of machine learning approaches in revealing complex feedback mechanisms within socio-ecological systems[^20^].

## Discussion

The DPSIR-A framework developed in this study, with the introduction of the Adaptability dimension, demonstrates strong theoretical applicability in assessing the resilience of tourism-oriented villages. Compared with traditional frameworks, it provides a more comprehensive representation of feedback mechanisms within social systems and allows for a closer integration of social and ecological processes. The results show that factors such as building disaster resistance and villagers’ disaster awareness play a dominant role in shaping resilience patterns. In Tibetan-Qiang settlements such as Jiuzhaigou, where cultural traditions remain deeply embedded, local construction practices and community cohesion function as informal buffers against natural hazards. This aligns with Gao et al.‘s findings on post-earthquake landscape resilience in Jiuzhaigou^34^. Furthermore, a comparison with previous research reveals that, unlike studies focusing solely on physical susceptibility, such as single geological factor assessments, this framework explains why some villages in high geological risk zones, such as parts of Ganhaizi, maintain high resilience levels. This is because high social adaptability can effectively offset physical exposure.

At the methodological level, the results further highlight the effectiveness of machine learning approaches in handling small and imbalanced disaster datasets in mountainous regions. The SMOTE-RF model performs particularly well under these conditions, improving the identification of low-resilience samples that are often misclassified by traditional methods. While models such as logistic regression or IVM tend to miss marginal cases, SMOTE enhances the representation of these samples through interpolation in feature space^13^. Beyond improving classification performance, the model also reveals nonlinear relationships among key variables. One notable finding is the dual role of road infrastructure. Roads increase exposure to geological hazards by disturbing slopes, yet they also provide essential support for evacuation and post-disaster recovery. This dual effect is difficult to capture using linear statistical approaches, but becomes evident in the random forest model. It highlights a form of resilience trade-off that is particularly relevant for infrastructure planning in mountainous areas^16,30^.

However, SMOTE operates in feature space rather than geographic space, so the synthetic samples it generates do not always correspond to realistic spatial configurations. This limitation is especially consequential in mountainous environments, where geological hazards are strongly clustered and locally specific. To reduce potential inconsistencies, physically unbuildable areas such as rivers and cliffs were excluded, spatial buffers were applied to reduce clustering effects, and five-fold cross-validation was used during model training. Even so, some uncertainty may remain. Future studies could adopt spatially explicit validation methods, including spatial block cross-validation and geographically constrained resampling, to further improve robustness.

A further consideration relates to the mismatch in spatial scale between the two types of indicators used in this study. High-resolution environmental variables originate as 30 m pixel data, while socioeconomic and survey-based indicators are collected at the village level and then uniformly assigned to every grid cell falling within each village boundary. Assigning values in this way creates a consistent spatial framework for machine learning, but it inevitably introduces a degree of scale generalization that smooths over spatial variation within individual villages. Future work could explore multi-scale modeling approaches that allow different types of data to be integrated at their native resolutions rather than forcing them into a single grid.

Building on the nonlinear mechanisms and spatial zoning results discussed above, this study further derives a set of planning and governance implications for resilience improvement. A key insight is the dual role of road infrastructure. In high mountain canyon regions such as Jiuzhaigou, road construction inevitably increases physical exposure to geological hazards due to slope cutting and terrain disturbance. At the same time, however, roads function as the primary lifeline for evacuation and post-disaster material transport, forming the physical foundation of recovery capacity.Evidence from the recovery process following the 2017 Jiuzhaigou Ms 7.0 earthquake further illustrates this point. High-resilience areas, such as the Ganhaizi Community, did not emerge simply from expanding infrastructure capacity, but were closely associated with targeted reinforcement and improved reliability of road systems. This suggests that when geological risks cannot be fully avoided, planning should focus less on avoiding exposure altogether and more on restructuring system balance through improved connectivity and structural stability.

Based on the spatial zoning results from the S-RF model, this study further recommends differentiated governance strategies tailored to each resilience level^29,37,41^. In high-resilience areas, particularly around the Ganhaizi Community, it would be effective to strengthen their role as regional support nodes by establishing disaster reduction command centers. These locations can take advantage of existing material reserves and communication capacity to provide support to surrounding low-resilience villages during emergencies. For medium-resilience areas, the priority lies in preventive reinforcement. This includes stabilizing road slopes and improving crowd management during peak tourist seasons, when increased visitor flows may intensify system pressure. Managing the transition from driving forces to pressure is especially important in such contexts.In contrast, extremely low-resilience areas, such as Erdaoqiao Village, require more restrictive and targeted interventions. Development intensity should be carefully controlled, and measures such as relocation or structural reinforcement need to be implemented in combination with continuous monitoring systems. Integrating these interventions with real-time warning technologies could further enhance risk management capacity.

The predictive performance of the S-RF model (AUC = 0.753) can be considered moderate but acceptable, given the limited number of historical hazard samples (*n* = 69) and the strong spatial heterogeneity of mountainous environments. Similar performance ranges (0.70–0.80) have been reported in previous studies of landslide and debris flow susceptibility under small-sample conditions^9,14,38^. More importantly, the value of the SMOTE-RF approach lies in its relative improvement. The increase in F1-score, particularly the 6.2% gain observed in this study, indicates a meaningful reduction in omission errors for low-resilience areas, which are often underrepresented in imbalanced datasets. This improvement enhances the practical reliability of the model in identifying high-risk locations.

Although the proposed framework improves the accuracy of village-level disaster resilience assessment, several limitations remain. These are mainly related to data constraints and the complexity of mountainous socio-ecological systems.

One important issue lies in the static nature of the current assessment. The analysis focuses on the potential resilience of villages under compound risks, rather than tracking how systems actually respond and recover after real hazard events^40^. This choice is largely driven by the lack of long-term post-disaster data in mountainous regions, where continuous monitoring of losses and recovery processes is difficult to obtain. As a result, the framework reflects the capacity of the system to cope with disturbances, but cannot fully verify whether high-resilience areas indeed experience lower losses or faster recovery. Future work could address this gap by integrating remote sensing time-series data and longitudinal socioeconomic observations, allowing resilience outcomes to be evaluated in a more dynamic manner.

The study also focuses on single-type geological hazards. However, under climate change, different hazards are increasingly interconnected. For example, extreme rainfall can trigger debris flows and further damage infrastructure. These cascading processes often lead to more severe impacts than single hazards. For such scenarios, the DPSIR-A framework requires further extension: the Pressure (P) dimension should incorporate inter-hazard triggering probabilities, the State (S) dimension should account for infrastructure interdependencies, and the Adaptability (A) dimension should reflect the comprehensive adaptive capacity of the regional emergency response network. Future research could integrate multi-hazard coupling models into this framework to identify the vulnerability transmission pathways of dominant factors under chained scenarios, thereby enabling the transition from single-hazard defense to integrated multi-hazard resilience management^31,38,42^.

Another issue concerns the balance between model performance and interpretability. While SMOTE was used to address the limited number of disaster samples and the random forest model demonstrated strong predictive performance, ensemble learning approaches still have limitations in terms of decision transparency. The random forest model provides a useful ranking of feature importance, but it does not clearly explain the marginal effects of individual variables. This limitation is particularly relevant for the Adaptability dimension, whose contribution to resilience outcomes cannot be fully interpreted within the current framework. Future research should incorporate interpretable machine learning methods, such as SHAP, to quantify the marginal contribution of each factor and to identify potential nonlinear threshold effects under different resilience levels. This would allow for a more transparent understanding of how socio-cultural adaptability interacts with environmental stressors in shaping resilience outcomes.

Finally, the representation of social factors is still relatively coarse. Some indicators are based on village-level survey data, which cannot capture differences within communities. This may limit the ability of the model to reflect variations in risk perception and behavior. Future research could use more detailed data sources, such as participatory mapping or location-based data, to better represent individual-level differences. Combining these data with environmental variables may provide a more comprehensive understanding of disaster resilience.

## Conclusion

This study, using zhangzha Town in Jiuzhaigou as a case, established a disaster-resilient assessment paradigm based on an improved DPSIR-A framework and multi-model machine learning. Key conclusions are as follows:

Framework Refinement and Application: The extended DPSIR-A model, through its explicit incorporation of the Adaptability (A) dimension, effectively captures the role of traditional ethnic ecological knowledge and community agency in mitigating geological risks in high-altitude tourism villages.

Model performance excellence: The SMOTE-enhanced random forest model demonstrated optimal predictive performance (AUC = 0.7526, Kappa = 0.754). This result highlights the strong robustness of ensemble learning methods when handling imbalanced geological hazard data.

Spatial heterogeneity is pronounced: Resilience in Zhangzha Town shows a distinctly uneven spatial pattern. High-resilience areas cluster in the socioeconomically developed northwestern plains, whereas low-resilience areas are constrained by topography and distributed along the eastern and southern margins.

Key driving factors: Terrain undulation acts as the baseline constraint for resilience. In contrast, building disaster resistance, villagers’ disaster awareness, and road distance are the key controllable factors enhancing resilience.

The methodology developed in this study is applicable not only to Jiuzhaigou but also provides a scientific template for precise disaster prevention and spatial resilience management in similar high-altitude, high-intensity seismic zones and tourism-oriented mountain villages worldwide. Enhancing model interpretability will be a key direction for future resilience assessment research.

## Data Availability

Data availabilityThe datasets generated during and/or analysed during the current study are available from the corresponding author on reasonable request.
